# How to properly use the PRISMA Statement

**DOI:** 10.1186/s13643-021-01671-z

**Published:** 2021-04-19

**Authors:** Rafael Sarkis-Onofre, Ferrán Catalá-López, Edoardo Aromataris, Craig Lockwood

**Affiliations:** 1 Graduate Program in Dentistry, Meridional Faculty, IMED, Passo Fundo, Brazil; 2 Department of Health Planning and Economics, National School of Public Health, Institute of Health Carlos III, Madrid, Spain; 3 Department of Medicine, University of Valencia/INCLIVA Health Research Institute and CIBERSAM, Valencia, Spain; 4 JBI, Faculty of Health and Medical Sciences, The University of Adelaide, Adelaide, Australia

## Editorial

It has been more than a decade since the original publication of the Preferred Reporting Items for Systematic Reviews and Meta-Analyses (PRISMA) Statement [1], and it has become one of the most cited reporting guidelines in biomedical literature [2, 3]. Since its publication, multiple extensions of the PRISMA Statement have been published concomitant with the advancement of knowledge synthesis methods [4–7]. The PRISMA2020 statement, an updated version has recently been published [8], and other extensions are currently in development [9].

The number of systematic reviews (SRs) has increased substantially over the past 20 years [10–12]. However, many SRs continue to be poorly conducted and reported [10, 11], and it is still common to see articles that use the PRISMA Statement and other reporting guidelines inappropriately, as was highlighted recently [13].

The PRISMA Statement and its extensions are an evidence-based, minimum set of recommendations designed primarily to encourage transparent and complete reporting of SRs. This growing set of guidelines have been developed to aid authors with appropriate reporting of different knowledge synthesis methods (such as SRs, scoping reviews, and review protocols) and to ensure that all aspects of this type of research are accurately and transparently reported. In other words, the PRISMA Statement is a road map to help authors best describe what was done, what was found, and in the case of a review protocol, what are they are planning to do.

Despite this clear and well-articulated intention [2–5], it is common for *Systematic Reviews* to receive manuscripts detailing the inappropriate use of the PRISMA Statement and its extensions. Most frequently, improper use appears with authors attempting to use the PRISMA statement as a methodological guideline for the design and conduct reviews, or identifying the PRISMA statement as a tool to assess the methodological quality of reviews, as seen in the following examples:

- “This scoping review will be conducted according to the Preferred Reporting Items for Systematic reviews and Meta-Analyses extension for Scoping Reviews (PRISMA-ScR) Statement.”
- “This protocol was designed based on the Preferred Reporting Items for Systematic Review and Meta-Analysis Protocols (PRISMA-P) Statement.”
- “The methodological quality of the included systematic reviews will be assessed with the Preferred Reporting Items for Systematic reviews and Meta-Analyses (PRISMA) Statement.”

Some organizations (such as Cochrane and JBI) have developed methodological guidelines that can help authors to design or conduct diverse types of knowledge synthesis rigorously [14, 15]. While the PRISMA statement is presented to predominantly guide reporting of a systematic review of interventions with meta-analyses, its detailed criteria can readily be applied to the majority of review types [13]. Differences between the role of the PRISMA Statement to guide reporting versus guidelines detailing methodological conduct is readily illustrated with the following example: the PRISMA Statement recommends that authors report their complete search strategies for all databases, registers, and websites (including any filters and limits used), but it does not include recommendations for designing and conducting literature searches [8]. If authors are interested in understanding how to create search strategies or which databases to include, they should refer to the methodological guidelines [12, 13]. Thus, the following examples can illustrate the appropriate use of the PRISMA Statement in research reporting:

- “The reporting of this systematic review was guided by the standards of the Preferred Reporting Items for Systematic Review and Meta-Analysis (PRISMA) Statement.”
- “This scoping review was reported according to the Preferred Reporting Items for Systematic Reviews and Meta-Analyses extension for Scoping Reviews (PRISMA-ScR).”
- “The protocol is being reported in accordance with the Preferred Reporting Items for Systematic Review and Meta-Analysis Protocols (PRISMA-P) Statement.”

*Systematic Reviews* supports the complete and transparent reporting of research. The Editors require the submission of a populated checklist from the relevant reporting guidelines, including the PRISMA checklist or the most appropriate PRISMA extension. Using the PRISMA statement and its extensions to write protocols or the completed review report, and completing the PRISMA checklists are likely to let reviewers and readers know what authors did and found, but also to optimize the quality of reporting and make the peer review process more efficient.

Transparent and complete reporting is an essential component of “good research”; it allows readers to judge key issues regarding the conduct of research and its trustworthiness and is also critical to establish a study’s replicability.

With the release of a major update to PRISMA in 2021, the appropriate use of the updated PRISMA Statement (and its extensions as those updates progress) will be an essential requirement for review based submissions, and we encourage authors, peer reviewers, and readers of *Systematic Reviews* to use and disseminate that initiative.

## Data Availability

We do not have any additional data or materials to share.

## Abbreviations

PRISMA: Preferred Reporting Items for Systematic Reviews and Meta-Analyses
PRISMA-ScR: Preferred Reporting Items for Systematic reviews and Meta-Analyses extension for Scoping Reviews
PRISMA-P: Preferred Reporting Items for Systematic Review and Meta-Analysis Protocols
SRs: Systematic reviews

## References

[1] Moher D, Liberati A, Tetzlaff J, Altman DG (2009). PRISMA Group. Preferred reporting items for systematic reviews and meta-analyses: the PRISMA statement. J Clin Epidemiol..

[2] Caulley L, Cheng W, Catala-Lopez F, Whelan J, Khoury M, Ferraro J (2020). Citation impact was highly variable for reporting guidelines of health research: a citation analysis. J Clin Epidemiol..

[3] Page MJ, Moher D (2017). Evaluations of the uptake and impact of the Preferred Reporting Items for Systematic Reviews and Meta-Analyses (PRISMA) Statement and extensions: a scoping review. Syst Rev..

[4] Rethlefsen ML, Kirtley S, Waffenschmidt S, Ayala AP, Moher D, Page MJ (2021). PRISMA-S: an extension to the PRISMA Statement for reporting literature searches in systematic reviews. Syst Rev..

[5] Tricco AC, Lillie E, Zarin W, O’Brien KK, Colquhoun H, Levac D (2018). PRISMA Extension for Scoping Reviews (PRISMA-ScR): Checklist and Explanation. Ann Intern Med..

[6] Moher D, Shamseer L, Clarke M, Ghersi D, Liberati A, Petticrew M (2015). Preferred reporting items for systematic review and meta-analysis protocols (PRISMA-P) 2015 statement. Syst Rev..

[7] Hutton B, Salanti G, Caldwell DM, Chaimani A, Schmid CH, Cameron C, Ioannidis JPA, Straus S, Thorlund K, Jansen JP, Mulrow C, Catalá-López F, Gøtzsche PC, Dickersin K, Boutron I, Altman DG, Moher D (2015). The PRISMA extension statement for reporting of systematic reviews incorporating network meta-analyses of health care interventions: checklist and explanations. Ann Intern Med..

[8] Page MJ, McKenzie JE, Bossuyt PM, Boutron I, Hoffmann TC, Mulrow CD, et al. The PRISMA 2020 statement: an updated guideline for reporting systematic reviews. Syst Rev. 2021;10(1):89. https://doi/10.1186/s13643-021-01626-4. 10.1186/s13643-021-01626-4 PMC8008539 33781348

[9] EQUATOR Network: Reporting guidelines under development for systematic reviews. https://www.equator-network.org/library/reporting-guidelines-under-development/reporting-guidelines-under-development-for-systematic-reviews/. Accessed 11 Feb 2021.

[10] Page MJ, Shamseer L, Altman DG, Tetzlaff J, Sampson M, Tricco AC, Catalá-López F, Li L, Reid EK, Sarkis-Onofre R, Moher D (2016). Epidemiology and Reporting Characteristics of Systematic Reviews of Biomedical Research: A Cross-Sectional Study. Plos Med..

[11] Ioannidis JP (2016). The Mass Production of Redundant, Misleading, and Conflicted Systematic Reviews and Meta-analyses. Milbank Q..

[12] Niforatos JD, Weaver M, Johansen ME. Assessment of Publication Trends of Systematic Reviews and Randomized Clinical Trials, 1995 to 2017. JAMA Intern Med. 2019;179(11):1593–4. https://doi.org/10.1001/jamainternmed.2019.3013. 10.1001/jamainternmed.2019.3013 PMC6664379 31355871

[13] Caulley L, Catala-Lopez F, Whelan J, Khoury M, Ferraro J, Cheng W (2020). Reporting guidelines of health research studies are frequently used inappropriately. J Clin Epidemiol..

[14] Higgins JPT, Thomas J, Chandler J, Cumpston M, Li T, Page MJ, Welch VA (editors). Cochrane Handbook for Systematic Reviews of Interventions. 2nd Edition ed. Chichester: Wiley; 2019.

[15] Aromataris E, Munn Z (Editors). JBI Manual for Evidence Synthesis. ed. Adelaide: JBI; 2020.

